# Self-reported quantity, compulsiveness and motives of exercise in patients with eating disorders and healthy controls: differences and similarities

**DOI:** 10.1186/s40337-018-0202-6

**Published:** 2018-07-01

**Authors:** Sandra Schlegl, Nina Dittmer, Svenja Hoffmann, Ulrich Voderholzer

**Affiliations:** 1 Department of Psychiatry and Psychotherapy, University Hospital of Munich (LMU), Nußbaumstraße 7, 80336 Munich, Germany; 2 Schoen Clinic Roseneck, Prien am Chiemsee, Germany; 3 Department of Clinical Psychology and Psychotherapy, Technical University of Dresden, Dresden, Germany; 4 Department of Counseling Psychology, University of Potsdam, Potsdam, Germany; 5 Department of Psychiatry and Psychotherapy, University Hospital of Freiburg, Freiburg im Breisgau, Germany

**Keywords:** Anorexia nervosa, Bulimia nervosa, Eating disorders, Compulsive exercise, Motives for exercise, Inpatient, Predictor, Healthy controls

## Abstract

**Background:**

Compulsive exercise (CE) is a frequent symptom in patients with eating disorders (EDs). It includes, in addition to quantitatively excessive exercise behaviour, a driven aspect and specific motives of exercise. CE is generally associated with worse therapy outcomes. The aims of the study were to compare self-reported quantity of exercise, compulsiveness of exercise as well as motives for exercise between patients with anorexia nervosa (AN), bulimia nervosa (BN) and healthy controls (HC). Additionally, we wanted to explore predictors of compulsive exercise (CE) in each group.

**Methods:**

We investigated 335 female participants (*n* = 226 inpatients, *n* = 109 HC) and assessed self-reported quantity of exercise, compulsiveness of exercise (Compulsive Exercise Test), motives for exercise (Exercise Motivations Inventory-2), ED symptoms (Eating Disorder Inventory-2), obsessive-compulsiveness (Obsessive-Compulsive Inventory-Revised), general psychopathology (Brief Symptom Inventory-18) and depression (Beck Depression Inventory-2).

**Results:**

Both patients with AN and BN exercised significantly more hours per week and showed significantly higher CE than HC; no differences were found between patients with AN and BN. Patients with EDs and HC also partly varied in motives for exercise. Specific motives were enjoyment, challenge, recognition and weight management in patients with EDs in contrast to ill-health avoidance and affiliation in HC. Patients with AN and BN only differed in regard to exercise for appearance reasons in which patients with BN scored higher. The most relevant predictor of CE across groups was exercise for weight and shape reasons.

**Conclusions:**

Exercise behaviours and motives differ between patients with EDs and HC. CE was pronounced in both patients with AN and BN. Therefore, future research should focus not only on CE in patients with AN, but also on CE in patients with BN. Similarities in CE in patients with AN and BN support a transdiagnostic approach during the development of interventions specifically targeting CE in patients with EDs.

**Electronic supplementary material:**

The online version of this article (10.1186/s40337-018-0202-6) contains supplementary material, which is available to authorized users.

## Plain English summary

Although exercise is part of a healthy lifestyle it can be detrimental to one’s health if it is practiced in a dysfunctional way. Persons who cannot bear not doing exercise and exercise even when injured or unwell show compulsive exercise (CE). This phenomenon is often present in persons suffering from anorexia nervosa (AN) or bulimia nervosa (BN). Our study compared exercise behaviours and motives for exercise of patients with AN and BN as well as healthy controls (HC). Results showed that patients with eating disorders (EDs) exercised more hours per week and showed higher CE than HC. A difference in CE between AN and BN could not be found. Furthermore, we wanted to find out what predicts CE. The most named reasons were weight and shape reasons. Patients with EDs exercise in a different way and pursue different goals compared to HC. CE was found in patients with AN and BN. Therefore, it is important to not only focus on patients with AN in future research. Similarities in CE in patients with AN and BN support the development of common interventions for the treatment of CE in patients with EDs.

## Background

Regular exercise is part of a healthy lifestyle and has positive effects on physical and mental health [1]. However, exercise can become dysfunctional. According to Adkins and Keel [2], there are two dimensions of dysfunctional exercise: the quantitative (“excessive”) dimension defined by frequency, intensity and duration of exercise as well as the qualitative (“compulsive”) dimension characterised by rigid exercise schedules, priority of exercise over other activities, record keeping, and feelings of guilt and anxiety when exercise sessions are missed. Previous studies revealed that 31 to 81% of patients with anorexia nervosa (AN) and 20 to 57% of patients with bulimia nervosa (BN) show dysfunctional exercise [3–8]. Dysfunctional exercise in eating disorders (EDs) is in general associated with worse therapy outcomes [9, 10], longer inpatient stays [11, 12], higher rates of inpatient dropout [13] and a higher risk of relapse and/or chronification [14, 15].

To date, several studies have investigated exercise behaviours and motives for exercise between samples of patients with mixed ED diagnoses and healthy controls (HC). Previous studies investigating differences in self-reported exercise quantity or objectively measured physical activity showed mixed findings. Some studies found higher means in patients with EDs compared to HC [13, 16–20], whereas others did not [16, 21–23]. Two studies even found higher scores in HC than in patients with EDs [13, 22]. A wide range of different questionnaires were used to assess dysfunctional exercise and reasons for exercise in patients with EDs and HC. In summary, patients showed higher scores on dysfunctional exercise than HC [16, 17, 20, 23–27]. Furthermore, study findings indicate that motivation or reasons for exercise differ between patients with EDs and HC. It was found that exercising for health reasons was less important in patients with EDs than in HC [16, 18]. Patients with EDs used exercise primarily for mood regulation [18, 20] or for reasons of weight, shape or physical attractiveness [20]. However, Boyd et al. [23] found no differences between patients with EDs and HC regarding exercise for mood and for weight control.

Apart from looking at patients with mixed EDs and HC, other studies directly compared patients with AN and BN. Findings indicate that self-reported quantity of exercise [5, 10, 20, 28, 29], objectively measured physical activity [18], questionnaire scores [24, 28, 30] as well as reasons for exercise [18] were comparable between patients with AN and BN.

One of the newest, most specific and comprehensive instruments for assessing compulsive exercise (CE), is the Compulsive Exercise Test (CET) [31]. The CET is based on the cognitive-behavioural maintenance model of excessive exercise. Two studies used the CET in a sample of patients with EDs and HC and found significantly higher scores in patients on the CET total score as well as on all subscales except for lack of exercise enjoyment and mood improvement [19, 32]. One study compared patients with AN, BN and EDs not otherwise specified (EDNOS) as well as HC and found that patients with AN scored lower than patients with BN on CET total score and on CET subscales except for exercise rigidity [33]. Besides, patients with AN did not differ from HC except for mood improvement. However, patients with BN differed from HC regarding CET total score as well as CET subscales avoidance and rule-driven behaviour and weight control exercise [33]. The only regression analysis for predicting CE in patients with EDs was done for patients with AN and revealed that it was significantly predicted by global ED psychopathology and anxiety, whereas depression and obsessive-compulsiveness were not significant predictors [34].

In summary, previous studies compared either samples of patients with mixed EDs to HC or only patients with AN to BN with regard to exercise behaviours and motives. However, only one study directly compared patients with AN, BN and HC which might contribute to a better understanding of how ED patients’ exercise behaviours and motives differ from HC. Furthermore, learning if and how the diagnostic subtypes of EDs differ might contribute to a better understanding of the psychopathology of these two disorders and might indicate whether transdiagnostic or diagnostic-specific treatment approaches to address CE in patients with EDs are needed. Additionally, so far only one study examined predictors of CE as assessed by the CET and this study included only patients with AN. Therefore, the aims of this study were 1) to further identify similarities and differences in exercise behaviours and motives in a sample with patients with AN, patients with BN and HC by directly comparing them and 2) to exploratively investigate predictors of CE in the different groups. We hypothesised that there were differences between patients with EDs and HC in quantity of exercise, in the extent of CE as well as in motives for exercise. We expected patients with EDs to show higher levels in all three domains compared to HC, but we did not expect differences between AN and BN.

## Methods

### Participants

We investigated a total of *N* = 335 participants: a consecutive sample of 226 female inpatients (all voluntary admissions) from 3 clinics with specialised units for EDs who were hospitalised between December 2012 and August 2013 and 109 HC who were primarily recruited from high schools and a university. Inclusion criteria for patients were a primary diagnosis of anorexia nervosa (F50.0/F50.1) or bulimia nervosa (F50.2/F50.3) according to ICD-10 (International Statistical Classification of Diseases and Related Health Problems-10) [35]. Patients were diagnosed by experienced ED clinicians during a routine intake interview. Furthermore, patients were to be aged between 13 and 60 years and to be female. Patients were excluded if their body-mass-index (BMI) was above 30 kg/m^2^ or greater than the 97^th^ percentile for adolescents. Inclusion criteria for HC were 1) female participants 2) age between 13 and 60 years 3) BMI between 18.5 kg/m^2^ and 25 kg/m^2^ for adults or a percentile between 10 and 90 for adolescents. Participants who reported 1) any bingeing, vomiting and/or laxative use 2) diet/low calorie food and/or excessive exercise twice or more a week 3) currently participating in competitive sports were excluded.

### Instruments

Patients filled in the following self-report measures:

#### Short Evaluation of Eating Disorders (SEED)

The SEED [36] is an instrument that assesses the three main symptoms for AN (degree of underweight, fear of weight gain and distortion of body perception) and BN (amount of binge eating, amount of compensatory behaviour and overconcern with body shape and weight). Each of the six items is answered on a 4-point Likert scale ranging from 0 (symptom not present) to 3 (symptom is extreme). The SEED was used to screen HC for eating disorders and for excluding them when indicated.

#### Self-reported exercise behaviour

A brief self-developed questionnaire was given to the participants to assess their exercise behaviour. It consisted of the following questions: Do you exercise regularly (if yes, how many hours per week)? What kind of exercise do you do and how frequently and how long? Do you Do you currently engage or have you participated in the past in competitive sports?

#### Compulsive Exercise Test (CET)

The CET [31] is a multidimensional measure designed to assess core factors operating in the maintenance of CE specifically among patients with EDs. It comprises 24 items and the following 5 subscales: avoidance and rule-driven behaviour, weight control exercise, mood improvement, lack of exercise enjoyment, and exercise rigidity. Subscales can be summed up to a CET total score. Ratings are based on a Likert scale ranging from 0 (never true) to 5 (always true). The German validation is currently underway (Schlegl S, Dittmer N, Vierl L, Rauh E, Huber T, Voderholzer U. Validation of the German version of the Compulsive Exercise Test in adolescent and adult eating disordered patients. In preparation). Cronbach’s α for the CET total score for this sample was .94 for AN, .92 for BN and .86 for HC. Cronbach’s α for the CET subscales for this sample ranged between .73 (exercise rigidity) and .95 (avoidance and rule-driven behaviour) for AN, between .77 (exercise rigidity) and .94 (avoidance and rule-driven behaviour) for BN and between .67 (exercise rigidity) and .86 (avoidance and rule-driven behaviour as well as mood improvement) for HC.

#### Exercise Motivations Inventory-2 (EMI-2)

The EMI-2 [37] offers a measure for the assessment of individuals’ motives for exercise. The EMI-2 is composed of 51 items and the following 14 subscales: stress management, revitalisation, enjoyment, challenge, social recognition, affiliation, competition, health pressures, ill-health avoidance, positive health, weight management, appearance, strength and endurance, and nimbleness. Ratings are given on a 6-point Likert scale ranging from 0 (not at all true) to 5 (very true). Cronbach’s α for the EMI-2 subscales for this sample ranged between .79 (revitalisation) and .96 (weight management) for AN, between .75 (revitalisation) and .93 (weight management) for BN and between .79 (ill-health avoidance) and .94 (nimbleness) for HC (apart from health pressure: .66, .45 and .61, respectively).

#### Eating Disorder Inventory-2 (EDI-2)

The EDI-2 [38] was used for the multidimensional assessment of the specific psychopathology of patients with EDs. It consists of 11 scales with 91 items that can be answered on a six-point scale from 1 (never) to 6 (always). Cronbach’s α for the EDI-2 total sum score for this sample was .96 for all three groups.

#### Obsessive-Compulsive Inventory-Revised (OCI-R)

The OCI-R [39] provides a brief assessment of the distress associated with six major obsessive-compulsive symptom domains (during the last month): checking, washing, ordering, hoarding, obsessing and neutralising. Each of these subscales contains 3 items that are rated on a 5-point Likert scale from 0 (not at all) to 4 (extremely). Cronbach’s α for the OCI-R total sum score for this sample was .91 for AN and BN and .87 for HC.

#### Beck Depression Inventory-2 (BDI-2)

The BDI-2 [40] is a self-rating instrument to assess the severity of depressive symptoms. Items can be rated on a four-point scale from 0 to 3 in terms of their occurrence and intensity during the last two weeks. Cronbach’s α for the BDI-2 sum total score for this sample was .91 for AN, .87 for BN and .89 for HC.

#### Brief Symptom Inventory-18 (BSI-18)

The BSI-18 [41] evaluates the general psychological distress level by means of 18 items belonging to three scales: somatisation, depression, and anxiety. Each item can be rated on a five-point scale from 0 (not at all) to 4 (extremely) in terms of the extent to which participants have been bothered by a symptom during the last week. Cronbach’s α for the BSI-18 total sum score for this sample was .91 for AN, .89 for BN and .88 for HC.

Sociodemographic variables and clinical variables (BMI, weight minimum, weight maximum) of patients were available from each patient’s clinical chart and self-reported by HC.

### Statistical analyses

To investigate differences between the three groups (AN, BN, HC) regarding sample description variables interval scale data were analysed using univariate analyses of variance followed by post hoc tests with Bonferroni correction; ordinal data were analysed using Kruskal-Wallis tests and nominal data using chi squared tests. To investigate differences between the three groups (AN, BN, HC) regarding compulsiveness of exercise and motives for exercise, multivariate analyses of variance (MANOVA) followed by post hoc tests with Bonferroni correction were done. Additionally, effect sizes (ES) (Cohen’s d) and 95% confidence intervals of ES were calculated for the pairwise comparisons of AN vs. BN, AN vs. HC and BN vs. HC. Furthermore, multivariate linear regression analyses (backward likelihood ratio method) with CET total score as the dependent variable were carried out (for AN, BN, HC separately) to examine predictors of CE in each group. In a first step, correlations were calculated between CET total score and quantitative (exercising hrs/week) and motivational aspects of exercise behaviour (EMI-2) as well as ED symptoms (EDI-2), depression (BDI-2), general psychopathology (BSI and OCI-R), sociodemographic and clinical variables. In a second step, multivariate regression analyses were performed with variables that were significantly correlated with CET total score as independent variables. All statistical analyses were performed with the ‘Statistical Package for Social Sciences’ (SPSS) for Windows, version 23.0.

## Results

A total of *N* = 384 participants filled in the questionnaires. However, *n* = 49 patients had to be excluded from analyses since they did not meet inclusion criteria. The final study sample consisted of *N* = 335 participants. Table 1 summarises the sociodemographic and clinical variables of the three groups. There were no differences between groups with regard to percentage of minors and adults, age or percentage of regular exercisers. However, groups differed with regard to education (χ^2^(2) = 19.02, *p* < .001), profession (χ^2^(2) =16.82, *p* < .001) and marital status (χ^2^(6) = 21.22, *p* = .002) with HC having a higher education and profession status and being less often single. Furthermore, they differed in BMI (F(2,332) = 375.39, *p* < .001, AN < BN, HC).

**Table 1:** Sample description (*N* = 335)

	Anorexia nervosa (*n* = 151)	Bulimia nervosa (*n* = 75)	Healthy controls (*n* = 109)
Age: M (SD)	21.11 (6.94)	23.09 (7.60)	23.23 (7.95)
Minor (%)	38.4%	25.3%	30.3%
Adult /%)	61.6%	74.7%	69.7%
Body-mass-index (kg/m^2^): M (SD)	15.23 (1.85)	20.90 (2.33)	20.98 (1.62)
Education (%)			
Higher education entrance qualification	68.2%	58.7%	90.8%
Intermediate school certificate	24.5%	25.3%	7.3%
Secondary general school certificate	4.0%	9.3%	0.9%
No graduation	2.0%	1.3%	–
Other graduation	1.3%	5.3%	0.9%
Profession (%)			
Still in school	41.9%	22.2%	33.6%
Still a university student	12.2%	13.9%	50.5%
Still in vocational training	11.5%	6.9%	0.9%
Finished vocational training	18.9%	26.4%	7.5%
University degree	6.1%	9.7%	4.7%
Without certification	8.8%	18.1%	2.8%
Other certification	0.7%	2.8%	–
Marital status (%)			
Single	76.8%	62.7%	52.3%
In relationship	17.2%	24.0%	36.7%
Married	4.6%	8.0%	9.2%
Other marital status	1.3%	5.3%	1.8%

### Self-reported quantity of exercise

60.9% of patients with AN, 68.0% of patients with BN and 67.9% of HC reported that they exercised on a regular basis. When including only those who exercise on a regular basis, the three groups significantly differed in exercising (hrs/week) (F(2,207) = 7.91, p < .001). Both patients with AN (M = 5.95, SD = 5.45) and BN (M = 6.24, SD = 3.85) exercised significantly more than HC (M = 3.60, SD = 2.42). ES [95% CI] was 0.06 [− 0.29; 0.40] for AN vs. BN, − 0.54 [− 0.86; − 0.22] for AN vs. HC and − 0.85 [− 1.23; − 0.48] for BN vs. HC.

### Compulsiveness of exercise

**Fig. 1:**
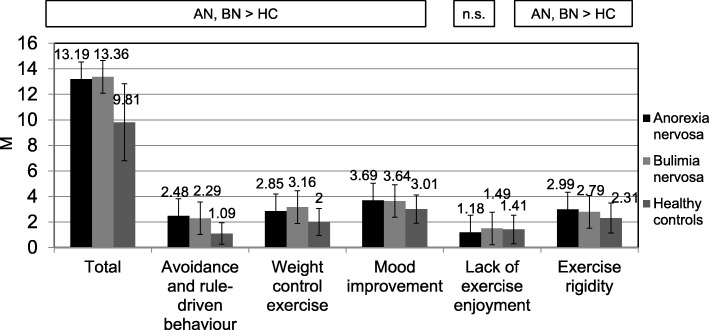
Differences in Compulsive Exercise Test scores between patients with anorexia nervosa (AN), bulimia nervosa (BN) and healthy controls (HC)

There were significant differences both between patients with AN and HC as well as between patients with BN and HC with regard to CET total score (high ES) and with regard to CET subscales avoidance and rule-driven behaviour (high ES), weight control exercise (moderate to high ES), mood improvement (moderate ES) and exercise rigidity (low to moderate ES), on which patients scored higher. Concerning lack of exercise enjoyment, no statistical difference was found between patients with AN and BN and HC. There were no significant differences between patients with AN and patients with BN, neither in CET total score nor in CET subscales (see Fig. 1). The statistical parameters of the MANOVA with regard to CET and the ES for the pairwise comparisons AN vs. BN, AN vs. HC, BN vs. HC can be found in Additional file 1: Table S1.

### Motives for exercise

**Fig. 2:**
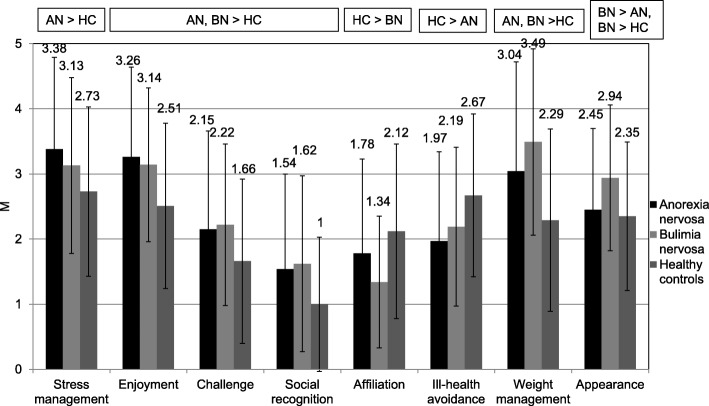
Significant differences in Exercise Motivations Inventory-2 scores between patients with anorexia nervosa (AN), bulimia nervosa (BN) and healthy controls (HC)

Patients with AN exercised significantly more to manage stress than HC. Both patient groups showed higher scores on enjoyment, challenge, social recognition and weight management compared to HC, whereas HC scored higher on ill-health avoidance than patients with AN and higher with regard to affiliation than patients with BN. Patients with AN and BN only differed in regard to exercise for appearance reasons in which patients with BN scored higher (see Fig. 2). Furthermore, patients with BN scored higher with regard to the latter motive compared to HC. ES were high with regard to weight management for patients with BN in comparison to HC, moderate with regard to enjoyment for both patients with AN and BN compared to HC, moderate with regard to social recognition, affiliation and appearance for patients with BN in comparison to HC as well as moderate with regard to ill-health avoidance for patients with AN in comparison to HC. All other significant comparisons showed only low ES.

No differences were found between the three groups for the following six motives: revitalisation, competition, health pressures, positive health, strength and endurance as well as nimbleness. Statistical parameters of the MANOVA with regard to EMI-2 as well as ES for the pairwise comparisons AN vs. BN, AN vs. HC, BN vs. HC are presented in Additional file 2: Table 2.

### Predictors of compulsive exercise (as measured by the CET total score)

Table 2 shows the variables included in the final model, separated for AN, BN and HC.

**Table 2:** Predictors of compulsive exercise (CET total score)

Significant predictors	Unstandardised coefficients		Standardised coefficients	t	p	95% confidence interval for B	
	B	SE	Beta			Lower boundary	Upper boundary
Anorexia nervosa (R^2^ = 83.6%)							
Weight management	1.90	.22	.68	8.56	.000	1.45	2.34
Enjoyment	1.02	.30	.23	3.36	.002	.41	1.63
Exercise hours per week	.15	.05	.22	3.24	.002	.06	.24
Maturity fears	.12	.04	.20	2.88	.006	.04	.20
Appearance	.67	.33	.16	2.05	.046	.012	1.34
Somatisation	−.71	.40	−.13	−1.79	.081	−1.51	.09
Impulse regulation	.07	.04	.12	1.69	.097	−.01	.16
Bulimia nervosa (R^2^ = 65.2%)							
Drive for thinness	.20	.08	.45	2.48	.020	.04	.36
Weight management	.94	.41	.35	2.29	.027	.11	1.77
Body dissatisfaction	−.09	.05	−.27	−1.79	.080	−.19	−.01
Challenge	.74	.28	.25	2.62	.012	.17	1.31
OCI-R Total	1.16	.51	.22	2.25	.029	.12	2.19
Healthy controls (R^2^ = 52.1%)							
Weight management	.88	.21	.41	4.30	.000	.47	1.29
Stress management	.61	.17	.27	3.53	.001	.27	.96
Drive for thinness	.12	.04	.26	2.76	.007	.03	.20
Strength and endurance	.43	.19	.17	2.20	.003	.04	.81

*CET* Compulsive Exercise Test, *OCI-R* Obsessive-Compulsive Inventory-Revised, *BDI-2* Beck Depression Inventory-2

## Discussion

In summary, we found several differences in exercise behaviours and motives between patients with EDs and HC whereas patients with AN and BN were very similar.

Both patients with AN and BN exercised more hours per week and showed higher CE than HC as measured by the CET. This is in line with previous studies that found that self-reported exercise quantity [16, 18–20] as well as CE assessed by other instruments than the CET [16, 17, 20, 23–27] were higher in patients with EDs than in HC. In accordance with Naylor et al. [19] who also used the CET, we found that patients with EDs and HC differed in all subscales of the CET except for lack of exercise enjoyment whereas Meyer et al. [32] found a significant difference in all subscales except for mood improvement. This inconsistency might be explained by different sample characteristics with Meyer et al. [32] including e.g. a high proportion of patients with EDNOS.

Furthermore, we found that the self-reported quantity of exercise in patients with AN and BN was comparable which is in line with previous studies [10, 12, 20, 28, 29]. Moreover, we found no differences in CE between the two diagnostic groups which is in accordance to previous findings that used other instruments [24, 28, 30], but contrary to the study from Sauchelli et al. [33] that also used the CET and found that patients with BN scored higher on the CET total score as well as on all subscales except for exercise rigidity. It is noticeable that the levels of CET total score and of CET subscales of patients with BN in both studies were relatively comparable whereas patients with AN scored considerably higher in our study. Despite both studies investigating inpatients, different sample characteristics such as a different age range, a lower BMI in patients with AN in our study or other unknown variables like illness duration or the proportion of subtype of AN might explain the contradictory results. Furthermore, the sample size of patients with AN in the study of Sauchelli et al. [33] was relatively small. Because of inconsistent results, there is an urgent need for further studies clarifying similarities or differences in CE in patients with AN compared to patients with BN.

Concerning motives for exercise, we found similarities and differences between patients with EDs and HC. Exercise for stress management and enjoyment were highest ranked in all groups. Significant differences between patients and HC were found for enjoyment, challenge, recognition and weight management with higher scores in patients and for ill-health avoidance as well as affiliation with higher scores in HC. Overall, HC showed more health-related motives than patients. This is in accordance with Markland and Ingledew [42] who found that positive health and ill-health avoidance were the most pronounced motives for exercise in HC when assessed with the EMI-2. Further studies using the Reasons for Exercise Inventory (REI) [43], also found that HC scored higher on health-related motives than patients with EDs [16, 18]. Mond and Calogero [20] found higher scores on the subscale weight control of the REI for patients with EDs than for HC which was also true for both patients with AN and BN in our study. Contrary to our results, in the study by Mond and Calogero [20] HC scored higher on the enjoyment subscale than patients with EDs. Patients with AN and BN did not differ in motives for exercise (except for appearance in which patients with BN scored higher) in our study. This result is in line with Bratland-Sanda et al. [18], who found no differences between patients with AN and BN on the REI subscales.

Our regression analyses revealed that exercise for weight and shape reasons was the strongest predictor of CE in all three groups. This is in line with a previous review that identified weight and shape concerns as one of the four key correlates of CE among patients with EDs [44]. In a previous study, exercising to improve mood was found to be the strongest predictor of driven exercise in patients with AN [16]. However, the authors argue that due to a lack of an association between physical activity and anxiety scores, the drive to exercise may not primarily be related to anxiety but instead be driven by body and weight concerns. Furthermore, Bratland-Sanda et al. [17] report that negative affect regulation was the most important explanatory factor for exercise dependence in patients as well as in HC, whereas weight/appearance was not a significant predictor. The authors state that motives for exercise might shift during phases of illness. They suggest that patients with EDs might be exercising more for weight and shape reasons in earlier phases of the illness while the more chronic ones might be exercising more for negative affect regulation. However, our data of inpatients suggest that severe and chronic cases might also show CE primarily for weight and shape reasons. In our regression analyses, exercise for affect regulation, a further key correlate of CE, was not a significant predictor in patients with either AN or BN. However, the EMI-2 might not have been an appropriate instrument to assess exercise for mood improvement or affect regulation since it only contains a subscale related to exercise for stress management. The REI also assesses exercise to cope with sadness, depression, anxiety and to improve one’s mood and might have been a better instrument. Compulsivity, a further key correlate of CE, turned out to be a significant predictor of CE for patients with BN, but not for patients with AN. This needs clarification and should be investigated in further studies. Perfectionism (EDI-2 subscale), the last key factor proposed in the maintaining model of CE did also not turn out to be a significant predictor in either group. In summary, our regression analyses provide preliminary information which factors might be essential in CE. But our results also highlight the need to empirically evaluate theoretical models of CE such as the model proposed by Meyer et al. [44].

Besides weight and shape reasons as shared predictors, specific predictors of CE that we found in our study were enjoyment and quantity of exercise in patients with AN. Higher enjoyment predicting higher CE might be explained by the fact that the more patients enjoy exercising, the more they might actually exercise and the higher the chance that exercise becomes driven. Quantity of exercise predicting higher CE is in line with studies that found a relationship between vigorous physical activity and exercise dependence [17] or between exercise frequency and CET total score [19] in mixed patients with EDs. Furthermore, challenge was a strong predictor in patients with BN. Challenge is seen as an intrinsic motive that satisfies the basic human need for competence [45]. This result suggests that the more patients with BN exercise for challenging themselves and the more they experience feelings of mastery and competence the higher the chance that exercise becomes driven. A specific predictor of CE in HC was stress management. This is in line with Pritchard and Beaver [46] who found that obligatory exercise was predicted by exercise for mood improvement in healthy women.

In summary, our results show that CE is a highly relevant symptom in both patients with AN and BN. Although CE is one of the most negative predictors of outcome and a positive predictor of chronification in patients with EDs, there is a lack of (evaluated) treatment approaches on how to manage these symptoms during therapy so far. Since studies found that changes in compulsive/excessive exercising were significantly associated with changes in BMI as well as in ED symptoms [5, 11, 30], interventions specifically targeting CE might be a way to improve outcomes.

### Strength and limitations of the study/future research

The study possesses several strengths: The sample size is high. Both patients with AN and BN as well as HC were assessed and directly compared. The CET, a multidimensional instrument for assessing CE, was used. Noetel et al. [34] favour the CET since it is an empirically derived and clinically sound measure of CE that avoids the shortcomings of previous studies categorizing CE according to arbitrary and inconsistent definitions. However, there are also several limitations of the study: First of all, all data except those for the BMI of patients were based on self-ratings, and no structured interview was conducted to assess ED diagnoses. Height and weight of HC were also only self-reported. Self-reported quantity of exercise could be biased: HC may claim to exercise more than is really the case, since activity is socially desirable. In patients with EDs, the opposite could be true. E.g. Alberti et al. [47] found that patients with AN underestimated their physical activity by subjective assessment when compared with objective measurement. So, accelerometers should be used in future studies. Second, all patients included were inpatients with severe and often long-standing symptomatology. Results may not be applicable to less severely impaired patients such as outpatients. Third, only females were included in the study. Future research should also address CE in men with EDs and compare them to women to explore possible gender differences. Forth, there is no psychometric validation of the German version of the EMI-2; investigating its psychometric properties is highly recommended. Fifth, the measures have different time frames ranging from one week to one month. Since the original instruments are intended to ask for certain defined time frames, we did not change time frames for our study. However, since the patients have a longstanding symptomatology symptoms and behaviours might not really differ if referred to the last week or the last month. Sixth, since the study had only a cross-sectional design it permitted only to assess simple associations, but not predictors. The direction of the associations or potential mediating variables should be investigated in future studies. Additionally, it would be interesting to evaluate exercise behaviours and motives for exercise in different stages of illness. Furthermore, investigating samples with only AN and BN patients that show CE e.g. diagnosed by an interview might contribute even more to a better understanding of differences in CE between patients with AN and BN. A structured interview for the assessment of CE in patients with EDs was recently suggested [48]. Further studies might also compare the two subtypes of AN as well as adolescents and adults.

## Conclusions

Exercise behaviours and motives of patients with EDs differ from HC. CE seems to be a highly relevant symptom in both patients with AN and BN. Therefore, future research on CE should focus not only on patients with AN, but also on patients with BN. Furthermore, there is an urge to develop and evaluate specific interventions to address CE in therapy. Our results suggest that CE presents similar in patients with AN and BN and therefore argue for a transdiagnostic approach with regard to the development of interventions specifically targeting CE in patients with EDs.

## Additional files

Additional file 1: **Table S1** Statistical parameters of the analysis of variance with subsequent post-hoc tests for the Compulsive Exercise Test (DOCX 22 kb)

Additional file 2: **Table S2**: Statistical parameters of the analysis of variance with subsequent post-hoc tests for the Exercise Motivations Inventory-2 (DOCX 28 kb)

## Abbreviations

AN: Anorexia nervosa
BDI-2: Beck Depression Inventory-2
BMI: Body-mass-index
BN: Bulimia nervosa
BSI-18: Brief Symptom Inventory-18
CE: Compulsive exercise
CET: Compulsive Exercise Test
ED: Eating disorder
EDI-2: Eating Disorder Inventory-2
EDNOS: Eating disorder not otherwise specified
EMI-2: Exercise Motivations Inventory-2
HC: Healthy controls
ICD-10: International Statistical Classification of Diseases and Related Health Problems-10
MANOVA: Multivariate analyses of variance
OCI-R: Obsessive-Compulsive Inventory-Revised; SPSS: Statistical Package for Social Sciences
