# Supplementary material for: Self-reported quantity, compulsiveness and motives of exercise in patients with eating disorders and healthy controls: differences and similarities
Source: J Eat Disord. 2018 Jul 1;6:17. doi: 10.1186/s40337-018-0202-6 (PMC6038234; doi:10.1186/s40337-018-0202-6)
Supplement: Supplementary file 1 — Table S1 Statistical parameters of the analysis of variance with subsequent post-hoc tests for the Compulsive Exercise Test (DOCX 22 kb) [file 40337_2018_202_MOESM1_ESM.docx]

Additional file 1: Table 1. Statistical parameters of the analysis of variance with subsequent post-hoc tests for the Compulsive Exercise Test

|  | **Anorexia nervosa** | **Bulimia nervosa** | **Healthy controls** | **MANOVA:**  **F (10, 654) = 9.41, p < .001** | | **Effect size**  **[95% CI]** | | |
| --- | --- | --- | --- | --- | --- | --- | --- | --- |
|  | M (SD) | M (SD) | M (SD) | ANOVA | Post-hoc | AN vs. BN | AN vs. HC | BN vs. HC |
| Total score | 13.19 (4.67) | 13.36 (3.86) | 9.81 (3.01) | F (2, 330) = 26.92, p < .001 | AN, BN > HC | 0.04  [-0.24, -0.32] | -0.83  [-1.09, -0.58] | -1.05  [-1.37, -0.74] |
| Avoidance and rule-driven behaviour | 2.48 (1.48) | 2.29 (1.35) | 1.09 (0.84) | F (2, 330) = 40.53, p < .001 | AN, BN > HC | -0.13  [-0.41, 0.15] | -1.11  [-1.38, -0.85] | -1.12  [-1.43, -0.80] |
| Weight control exercise | 2.85 (1.47) | 3.16 (1.29) | 2.00 (1.06) | F (2, 330) = 20.84, p < .001 | AN, BN > HC | 0.22  [-0.06, 0.50] | -0.65  [-0.90, -0.39] | -1.00  [-1.31, -0.69] |
| Mood improvement | 3.69 (1.13) | 3.64 (1.03) | 3.01 (1.11) | F (2, 330) = 13.33, p < .001 | AN, BN > HC | -0.05  [-0.32, 0.23] | -0.61  [-0.86, -0.35] | -0.58  [-0.89, -0.28] |
| Lack of exercise enjoyment | 1.18 (1.04) | 1.49 (1.06) | 1.41 (1.12) | F (2, 330) = 2.51, p < .083 | -- | 0.30  [0.02, 0.58] | 0.21  [-0.03, 0.46] | -0.07  [-0.37, 0.22] |
| Exercise rigidity | 2.99 (1.34) | 2.79 (1.28) | 2.31 (1.17) | F (2, 330) = 9.20, p < .001 | AN, BN > HC | -0.15  [-0.43, 0.13] | -0.54  [-0.79, -0.28] | -0.40  [-0.69, -0.10] |

Notes: AN = Anorexia nervosa, BN = Bulimia nervosa, HC = Healthy controls, CI = Confidence interval.
