# Supplementary material for: Self-reported quantity, compulsiveness and motives of exercise in patients with eating disorders and healthy controls: differences and similarities
Source: J Eat Disord. 2018 Jul 1;6:17. doi: 10.1186/s40337-018-0202-6 (PMC6038234; doi:10.1186/s40337-018-0202-6)
Supplement: Supplementary file 2 — Table S2: Statistical parameters of the analysis of variance with subsequent post-hoc tests for the Exercise Motivations Inventory-2 (DOCX 28 kb) [file 40337_2018_202_MOESM2_ESM.docx]

Additional file 1: Table S2. Statistical parameters of the analysis of variance with subsequent post-hoc tests for the Exercise Motivations Inventory-2

|  | **Anorexia nervosa** | **Bulimia nervosa** | **Healthy controls** | **MANOVA:**  F (28, 550) = 5.27, p < .001 | | **Effect size**  **[95% CI]** | | |
| --- | --- | --- | --- | --- | --- | --- | --- | --- |
|  | M (SD) | M (SD) | M (SD) | ANOVA | Post-hoc | AN vs. BN | AN vs. HC | BN vs. HC |
| STR | 3.38 (1.41) | 3.13 (1.35) | 2.73 (1.30) | F (2, 287) = 6.19, p = .002 | AN > HC | -0.18  [-0.48, 0.12] | -0.48  [-0.75, -0.21] | -0.30  [-0.61, 0.01] |
| REV | 3.42 (1.22) | 3.03 (1.13) | 3.11 (1.24) | F (2, 287) = 2.89, p = .057 | -- | -0.33  [-0.63, -0.03] | -0.25  [-0.52, -0.01] | 0.07  [-0.24, 0.37] |
| ENJ | 3.26 (1.38) | 3.14 (1.18) | 2.51 (1.27) | F (2, 287) = 9.99, p < .001 | AN, BN > HC | -0.09  [-0.39, 0.21] | -0.56  [-0.83, -0.30] | -0.51  [-0.82, -0.20] |
| CHA | 2.15 (1.51) | 2.22 (1.24) | 1.66 (1.26) | F (2, 287) = 4.74, p = .009 | -- | 0.05  [-0.25, -0.35] | -0.35  [-0.62, -0.08] | -0.45  [-0.76, -0.14] |
| REC | 1.54 (1.46) | 1.62 (1.35) | 1.00 (1.03) | F (2, 287) = 6.45, p = .002 | AN, BN > HC | 0.06  [-0.24, -0.35] | -0.42  [-0.69, -0.16] | -0.53  [-0.84, -0.22] |
| AFF | 1.78 (1.45) | 1.34 (1.01) | 2.12 (1.34) | F (2, 287) = 7.12, p < .001 | HC > AN, BN | -0.34  [-0.64, -0.04] | 0.24  [-0.02, 0.51] | 0.64  [0.33, 0.95] |
| COM | 1.20 (1.34) | 0.95 (1.19) | 0.93 (0.97) | F (2, 287) = 1.74, p = .178 | -- | -0.19  [-0.49, 0.10] | -0.23  [-0.49, 0.04] | -0.02  [-0.33, 0.29] |
| HPR | 0.47 (0.80) | 0.56 (0.81) | 0.70 (0.89) | F (2, 287) = 2.03, p = .133 | HC > AN | 0.11  [-0.19, 0.41] | 0.27  [0.01, 0.54] | 0.16  [-0.14, 0.47] |
| ILL | 1.97 (1.37) | 2.19 (1.22) | 2.67 (1.25) | F (2, 287) = 8.16, p < .001 | HC > AN, BN | 0.17  [-0.13, 0.47] | 0.53  [0.26, 0.80] | 0.39  [0.08, 0.70] |
| POS | 3.16 (1.45) | 3.22 (1.31) | 3.47 (1.16) | F (2, 287) = 1.68, p = .188 | -- | 0.04  [-0.26, 0.34] | 0.23  [-0.03, 0.50] | 0.21  [-0.10, 0.51] |
| WEI | 3.04 (1.68) | 3.49 (1.43) | 2.29 (1.40) | F (2, 287) = 13.80, p < .001 | BN > HC | 0.28  [-0.02, 0.59] | -0.48  [-0.75, -0.21] | -0.85  [-1.17, -0.53] |
| APP | 2.45 (1.25) | 2.94 (1.12) | 2.35 (1.14) | F (2, 287) = 5.62, p = .004 | BN > AN | 0.41  [0.11, 0,71] | -0.08  [-0.35, 0.18] | -0.52  [-0.83, -0.21] |
| SAE | 2.91 (1.46) | 3.18 (1.15) | 2.89 (1.19) | F (2, 287) = 1.19, p = .306 | -- | 0.20  [-0.10; 0.50] | -0.02  [-0.28, 0.25] | -0.25  [-0.56, 0.06] |
| NIM | 2.90 (1.63) | 2.71 (1.41) | 2.95 (1.34) | F (2, 287) = 0.60, p = .550 | -- | -0.12  [-0.42, 0.18] | 0.03  [-0.23, 0.30] | 0.18  [-0.13, 0.48] |

Notes: AN = Anorexia nervosa, BN = Bulimia nervosa, HC = Healthy controls; subscales of the Exercise Motivations Inventory-2: STR = Stress management, REV = Revitalisation, ENJ = Enjoyment, CHA = Challenge, REC = Social recognition, AFF = Affiliation, COM = Competition, HPR = Health pressures, ILL = Ill-health avoidance, POS = Positive health, WEI = Weight management, APP = Appearance, SAE = Strength and endurance, NIM = Nimbleness, CI = Confidence interval.
